# The Changes in the Shapes of Teeth That Are Necesary or Proper for the Treatment of Decay

**Published:** 1874-10

**Authors:** Edmund Noyes

**Affiliations:** Chicago.


### ARTICLE II.

### *The Changes in the Shapes of Teeth that are Necessary or Proper for the Treatment of Decay.*

BY EDMUND NOYES, CHICAGO.

Read before the Illinois State Dental Society.

No one having much opportunity to observe teeth which have been operated upon, can have failed to perceive that this subject is one of great practical importance, and also that in very numerous instances it has received no attention, or else a mistake has been made in the treatment, so that in many cases of refilling the previous improper cutting greatly increases the difficulty of making successful operations.

It may be asked at the outset whether nature is not the best judge of what the shapes of teeth should be, and therefore why we venture to make any changes of shape at all.

Nature is doubtless perfect in her construction whenever she is able to work up to her perfect ideal, and from her best specimens we shall learn the principles that will guide us, and find the models toward which we must work in all the changes which we venture to make in the forms of teeth, but we find nature herself adopting so many different forms, and some of them so much more liable to decay than others, that we cannot suppose each of them to be the most perfect possible for the individual case, when we have regard merely to capability of resisting decay. In most cases, however, we are not called upon to decide whether *any*

changes of form shall be made or not, for we find them already made by the progress of decay, and these changes are often very important, even though the amount of substance removed is very little, so that the usual question for us is not whether the form of the tooth shall be changed or not, but whether we shall carefully and intelligently change its form in such way as will diminish its liability to future decay, or whether we shall incidentally or carelessly allow changes which will often increase its liability to decay beyond what it had as nature first formed it.

As already intimated, we must seek for general principles and models by a study of forms and arrangement nature has adopted in the dentures which we find least subject to decay. We shall find such teeth convex in every part, (outside the alveolus,) except the grinding surfaces, and with what may be described as bell-shaped crowns.

The greatest circumference being but a short distance removed from the grinding surface toward the gum, and this, of course, determines their points of contact.

The arched arrangement of the teeth in the jaw would lead us to expect them to be compressed toward the palate so that the lingual side would present less surface than the buccal, and we find this compression even greater than the convexity of the arch requires, so that the points of contact are nearer to the buccal than to the palatal side of the teeth. I think this characteristic is more frequent and perhaps more marked in the upper teeth than in the lower ones.

We find (in these perfect dentures) that the surfaces in contact are convex, so that it is more proper to say *points* of contact, than *surfaces* of contact, though unfortunately that is not so often the case after filling unless some care is taken to make it so. Of course these perfect teeth separate from each other in every direction from their points of contact, very slightly toward the grinding surface, somewhat more toward the gum, and often most of all toward the palate (or tongue.)

Whenever teeth are cut in operating upon them we almost of necessity leave nearly plane surfaces, and the obvious inference from the form the best teeth possess naturally, is that what we cut away should be cut in several different planes, and so that the surfaces of adjoining teeth shall always be divergent from each other.

I presume all will agree that molars and bicuspids decayed upon their proximal surfaces, presents greater difficulties in the way of making permanently successful operations, than do any other teeth, and certainly it is more difficult to make changes in the shapes of them that will avail to stop the progress of incipient decay, or will diminish the liability to recurrence of decay after filliug, than is the case with other teeth.

When they are regular in position and reasonably well formed, if the cavities are small or only incipient I think the best results are obtained by leaving the points of contact as nearly as possible untouched (unless they require to be moved a little toward the gum,) and slightly opening several of the divergent spaces or Vs, usually the one toward the grinding surface slightly, and the one toward the palate more, so as to leave a more considerable space in that direction than any other, except that in lower teeth it is often better to make the larger opening toward the cheek instead of toward the tongue. It is sometimes possible also, and often very desirable, to enlarge the V space toward the alveolus, but it should very seldom, if ever, be done to such an extent as to shoulder the neck of the tooth.

If decay has made great progress the relative strength of the remaining portions of the teeth may have determining influence as to the position of the V spaces after the fillings are completed. (The question of restoring contour will have its influence also,) but in no case, when teeth stand close together, should fillings be finished up with parallel plane surfaces opposed to each other, whether in actual contact or not, and whether the surfaces are gold, or dentine, or enamel, and the difficulty of following this rule in certain cases, should only make us the more careful to do so.

In no case should *any* change be made in the shapes of teeth without first carefully observing their natural form, the situation and extent of the cavities in them, their position as regards adjoining teeth, and especially their articulation, or occlusion with opposing teeth, so as to foresee whether if isolated they will remain so, or whether the forces acting upon them will twist them so as to make surfaces now divergent after a time become parallel.

Such observation will often make us deviate from the usual plan of making the largest space toward the palate above or toward the cheek below. Crowding or irregularity of the teeth in any part of the mouth will very often greatly increase the difficulty of making any changes of form that will be of practical use in promoting their preservation, without cutting them so much as seriously to injure their appearance or usefulness, and the ordinary rules will often require to be modified or reversed to suit the requirements of such cases, while the general *principle* always to leave divergent surfaces as little as possible in contact, is of almost universal application.

There are probably many cases, when nearly all the proximal surfaces have begun to decay, in which it is best to isolate all the teeth by permanent seperations, and this will often be accomplished incidentally without special reference to it, in carrying out the principles and rules already suggested.

These permanent separations should almost always be *very slight* at the points of nearest approach, too little in most cases (when there is but little decay,) to admit of proper manipulation without wedging before operating. I usually resort to slow wedging, finding it practically impossible *for me* to get space enough to work in by quick wedging; a permanent space half the thickness of Froid's file No. 0, is often enough *at the point of nearest approach*, but the proper manipulation of the surfaces requires at the time of operating a space as great as the thickness of Froid's No. 2, and it is often better that it should be considerably greater than that.

In many cases permanent isolation, whether desirable or not, is practically impossible, and in a large proportion of cases we must do all our cutting with as distinct pre-vision as possible, of what the conditions and consequences will be if the teeth come again into actual contact. I desire *specially*, to avoid giving the impression that a great *amount* of cutting is often necessary, and to give great emphasis to the fact that it is often more important to *avoid* cutting away the tooth substance that needs to remain, than it is to cut what needs to be removed; we often find that the separating file has removed the most prominent parts of the proximal surfaces in actual contact, much greater in extent than the one nature had originally provided. Such teeth will more surely decay the second time than they did the first, so far as decay is promoted by shape merely.

I think that all necessary changes of form may be made in most cases with only slight diminution of the grinding surfaces, and often I think, without removing quite all the enamel at any point.

The work can be done with any appropiate cutting instrument, usually with files, or chisels followed by files; it is essential that the cut surfaces should be finished with faces that will receive the action of polishing material upon every part of them, and this is often easier obtained with files, and strips of corundum tape or their equivalent, than it is with chisels alone.

The "hard-bits" made by S. S. White, are very useful for the first cutting, but there are many places for which they are too thick, and a straight chisel no thicker than a separating file must be used.

When separating files are used the saw edge should be bevelled or removed on the grindstone so as to avoid all danger of scratching the adjoining tooth, for cutting in divergent planes make it impossible to cut but one tooth at a time. The cutting of each tooth should be in two and sometimes three different planes, all divering from what is intended to be the point of contact; and these points of contact in adjoining teeth should of course be made so as to oppose each other; in other words, we must take care to leave most prominent points which we desire to come in contact.

After the file, in finishing the fillings or cut surfaces, or both must come the corundum tape or its equivalent, pumice stone on tape or stick (tape if possible,) oxide of tin in the same way, or anything that will perfectly obliterate the file marks, and put upon the best possible polish. A good polish is absolutely indispensible to every proximal surface operated upon.

The incisor and cuspid teeth present far less difficulties than the bicuspid and molars. They are much easier to isolate permanently, and are easier preserved without perfect isolation. If regular and well formed they can almost always be cut away enough toward the palate surface without changing their appearance in front, but even these, in some cases of crowding and overlapping, will tax skill and good judgment to the very utmost in order to make operations that will stand the test of time, either with or without changing their shape.

It is sometimes objected to cutting away the front teeth toward the palate side, that it removes the fold or ridge of enamel at the side of the tooth, and so weakens it and impairs its usefulness for grinding food, and in some instances this may have influence in determining our treatment, but in most cases this fold of enamel is not prominent enough to require any change of the plan of treatment on account of it, and sometimes it had doubtless better be sacrificed than to leave the teeth exposed to recurrence of decay.

We sometimes find teeth with a hollow or longitudinal furrow upon the proximal faces, so that the cervical wall of a cavity in such a tooth will have a notch in it. This can often be obliterated without shouldering the neck of the tooth, by enlarging the V space toward the gum; if that is impossible, we should make as near approach to it as we can, and leave the groove as broad and shallow as possible, and then the greatest care must be taken to finish down the cervical margin of the filling so as not to leave it overlapping or projecting; the separating file will of course cut down to the tooth near the buccal and palatal side and leaves the hollow untouched; if left so, we may feel sure of renewed decay sometime, at the cervical margin of that filling.

It needs to be said here (as well as always when the preservation of teeth is the subject of discussion,) that careful and constant attention to cleanliness is indispensable to the success of any operation upon the proximal surfaces of teeth; and one principal object to be kept in view in pursuing the line of treatment indicated in this paper, is to give to the tooth pick, the floss silk, and the brush a fair opportunity.

